# Simultaneous determination of 24 opioids, stimulants and new psychoactive substances in wastewater

**DOI:** 10.1016/j.mex.2019.04.016

**Published:** 2019-04-19

**Authors:** Richard Bade, Maulik Ghetia, Lynn Nguyen, Benjamin J. Tscharke, Jason M. White, Cobus Gerber

**Affiliations:** a School of Pharmacy and Medical Sciences, University of South Australia, Adelaide, South Australia, 5000, Australia; b Queensland Alliance for Environmental Health Science (QAEHS), University of Queensland, 20 Cornwall Street Woolloongabba, Queensland, 4102, Australia

**Keywords:** A quantitative method for the analysis of opioids, Stimulants and new psychoactive substances in wastewater, Triple quadrupole, Matrix effects, Synthetic cathinones, Mass spectrometry, Illicit drugs

## Abstract

Wastewater-based epidemiology has become a reputable means to estimate drug consumption within a community. However, these methods typically focus solely on illicit drugs or a single chemical family, with multi-class methods out of favour due to the increased analytical challenges.

• A sensitive liquid chromatography – mass spectrometry method was developed for the simultaneous determination of 24 opioids, stimulants and new psychoactive substances in influent wastewater.
• Filtered wastewater samples, preserved with sodium metabisulfite, were pretreated and 1000 times concentrated using off-line solid phase extraction.
• The method was optimised and fully validated for all compounds, with limits of quantification between 0.2 and 300 ng/L.

## Graphical abstract

**Figure:**
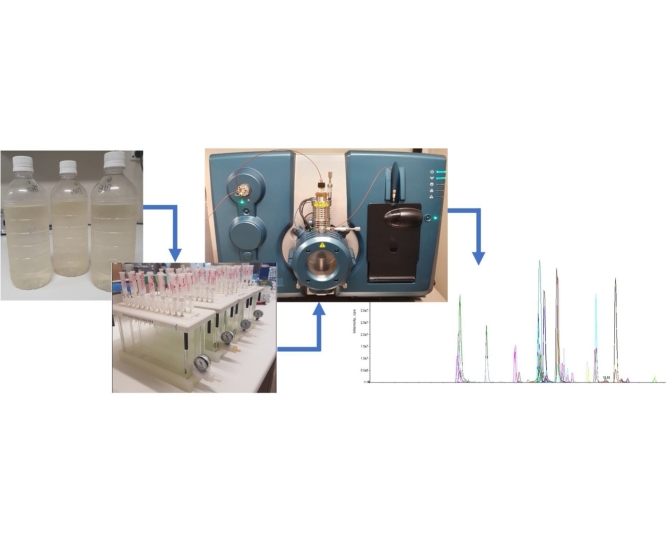

**Specifications Table**

**Table:** 

Subject Area:	*Chemistry*
More specific subject area:	*Analytical chemistry/ wastewater-based epidemiology*
Method name:	*A quantitative method for the analysis of opioids, stimulants and new psychoactive substances in wastewater*
Name and reference of original method:	*Irvine (2011)*: [1] *Tscharke (2016)*: [2]
Resource availability:	*NA*

## Method details

The current method focuses on the quantitative determination of 24 opioids, stimulants and new psychoactive substances and builds on methods previously published [1,2]. The compounds for this work were selected to cover a range of chemicals currently of wastewater-based epidemiology interest and health concern. The illicit drugs included in this study represent those of highest (inter)national consumption and currently monitored as part of the international multi-laboratory exercise, organised by the Sewage Core Europe (SCorE) group, in which our group takes part. This exercise focuses on the illicit drugs amphetamine, heroin, methamphetamine and MDMA [3]. All of these illicit drugs are also important from a health standpoint and were included in this study as the parent drug or metabolite, along with the methamphetamine-specific biomarker pholedrine. Alongside these illicit drugs, eight new psychoactive substances (NPS) were also included (3-trifluoromethylphenylpiperazine (TFMPP), 3,4-methylenedioxyamphetamine (MDA), alpha PVP, ethylone, mephedrone, methcathinone, methylenedioxypyrovalerone (MDPV), methylone and para-methoxyamphetamine (PMA)). NPS are of global concern, with mephedrone one of the most common synthetic cathinones available globally [4], while ethylone, methylone, MDPV and alpha PVP have previously been found in wastewater samples [[5], [6], [7], [8], [9], [10]].

The nicotine metabolite cotinine has previously been utilised to estimate community smoking rates and has thus also been included in this study [11,12]. Five parent pharmaceutical opioids and/or their metabolites (buprenorphine and norbuprenorphine, codeine, fentanyl (as norfentanyl), methadone, morphine and oxycodone (as noroxycodone) were also analysed.

The sensitive analytical method herein thus encompasses many areas of current health concern – illicit drugs, NPS, tobacco use and opioid consumption and allows a more comprehensive overview of drug consumption in a community in a timely manner.

### Chemicals and materials

3,4-methylenedioxyamphetamine (MDA), 3,4-methylenedioxymethamphetamine (MDMA), 3-trifluoromethylphenylpiperazine (TFMPP), 6-monoacetylmorphine (6-MAM), alpha PVP, amphetamine, benzoylecgonine, buprenorphine, cocaine, codeine, cotinine, ethylone, methylenedioxypyrovalerone (MDPV), mephedrone, methadone, methamphetamine, methcathinone, methylone, morphine, norbuprenorphine, norfentanyl, noroxycodone, pholedrine and para-methoxyamphetamine (PMA), were purchased as certified solutions or powdered salts from Cerilliant (Round Rock, USA) or Toronto Research Chemicals (Toronto, Canada). The isotopically-labelled internal standards (ILIS): alpha PVP-d_8_, amphetamine-d_5_, benzoylecgonine-d_3_, buprenorphine-d_3_, cocaine-d_3_, codeine-d_3_, cotinine-d_3_, hydroxycotinine-d_3_, 6-monoacetylmorphine-d_3_ (6-MAM-d_3_), 3,4-methylenedioxyamphetamine-d_5_ (MDA-d_5_), 3,4-methylenedioxymethamphetamine-d_5_ (MDMA-d_5_), methylenedioxypyrovalerone-d_8_ (MDPV-d_8_), mephedrone-d_3_, methadone-d_3_, methamphetamine-d_5_, methcathinone-d_3_, methylone-d_3_, morphine-d_3_, norbuprenorphine-d_3_, norfentanyl-d_3_, noroxycodone-d_3_, pholedrine-d_3_ and 3-trifluoromethylphenylpiperazine-d_8_ (TFMPP-d_8_) were purchased from Cerilliant (Round Rock, USA) or Toronto Research Chemicals (Toronto, Canada). Sodium acetate and ammonium formate were purchased from VWR Chemicals (Tingalpa, Queensland, Australia); acetonitrile, methanol, isopropanol, dichloromethane, glacial acetic acid, ammonia (28%) and formic acid (98–100%) from Thermo Fischer Scientific Australia (Scoresby, VIC, Australia) while hydrochloric acid (37%) and sodium metabisulfite (Na_2_S_2_O_5_) were purchased from Chem-Supply (Gillman, SA, Australia) Ultrapure water was prepared using an Arium® pro VF system (Sartorius Stedim biotech).

### Samples

The method was validated using “grab samples” of influent wastewater collected from a wastewater treatment plant (WWTP) in South Australia. All samples were collected in high density polyethylene terephthalate (PET) bottles, preserved with sodium metabisulfite (0.5% m/v), and stored onsite at 4 °C until all samples of the week had been collected, before being transported to our laboratory where they were immediately filtered under vacuum using two glass microfibre filter papers for each sample (GF/A 1.6 mm, Whatman, Kent, U.K.), then stored at 4 °C prior to sample treatment. The containers were tested to ensure no losses for any of the analytes.

### Sample treatment

Filtered samples (100 mL) were warmed to room temperature and, if needed, acetic acid (10%) was added to adjust the pH of the samples to 4.5–5. Mixed internal standard (100 μL of 50 μg/L) was then added to all samples. The acidified samples were loaded onto mixed mode (reversed phase and ion exchange) UCT XRADH 506 SPE cartridges (UCT Inc., Bristol, PA, USA); 500 mg/6 mL) which had been conditioned with methanol (6 mL) and sodium acetate buffer (20 mM pH 5, 6 mL). The cartridges were washed with sodium acetate buffer (6 mL), 0.1 M acetic acid (2 mL) and methanol (6 mL). Analytes were then eluted with a mixture of dichloromethane:isopropanol:ammonia (80:16:4, 6 mL)and evaporated to 200 μL under nitrogen at 40 °C, when 1% hydrochloric acid in methanol was added, then evaporated to dryness. The dry residue was reconstituted with 0.1% formic acid in methanol (20 μL) and 0.1% formic acid in milliQ water (80 μL) to give a final concentration factor of 1000 times.

Analyses were performed by injecting 2 μL of the final extract in the UPLC-MS/MS system.

### Instrumentation

LC–MS analyses were conducted using a Sciex ExionLC coupled to a Sciex 6500+ QTrap (Toronto, Canada), fitted with a TurboSpray IonDrive source. The chromatographic separation was carried out using a Kinetex Biphenyl column (150 × 2.1 mm × 1.7 μm) connected to a Biphenyl guard column (SecurityGuard ULTRA; 4 × 2.0 mm; Phenomenex Inc., Torrance, CA) at a flow rate of 0.3 mL min^−1^ with a 2 μL injection volume. The mobile phases used were 95% water with 5% methanol and 0.1% formic acid (solvent A) and 95% methanol with 5% water and 0.1% formic acid (solvent B). The initial percentage of B was 2% and after 2 min was linearly increased to 100% over 16 min, followed by a 1 min isocratic period, then returned to initial conditions in 0.1 min and remained steady for the final 2.9 min. The total run time was 20 min.

The ion source parameters were as follows: 500 °C; curtain gas, 25; collision gas, high; ion spray voltage, 5500 V; ion source gas 1 and ion source gas 2, 50. Mass spectrometric analyses were performed in positive mode using multiple reaction monitoring (MRM) using two of the most abundant transitions of the precursor ion and one for the deuterated analogues (Table 1).

**Table 1:** Mass spectrometric parameters for all compounds.

Compound	Internal Standard	Precursor ion ([M+H]^+^)	Product Ions^a^	Declustering Potential (V)	Collision Energy (V)
6-MAM	6-MAM-d_3_	328.3	165.2	110	35
			211.0	110	35
Alpha PVP	Alpha PVP-d_8_	232.3	91.3	50	25
			77.2	50	50
Amphetamine	Amphetamine-d_5_	136.1	119.1	20	14
			90.6	20	22
Benzoylecgonine	Benzoylecgonine-d_3_	290.1	105.1	50	41
			77.0	50	54
Buprenorphine	Buprenorphine-d_4_	468.4	396.3	150	50
			414.3	150	50
Cocaine	Cocaine-d_3_	304.3	182.2	50	38
			105.1	50	25
Codeine	Codeine-d_3_	300.1	165.2	80	54
			153.2	80	54
Cotinine	Cotinine-d_3_	177.1	81.0	50	27
			98.1	50	31
Ethylone	Methylone-d_3_	222.3	174.5	50	29
			146.2	50	22
MDA	MDA-d_5_	180.1	163.1	20	14
			105.1	20	25
MDMA	MDMA-d_5_	194.1	163.1	20	16
			105.1	20	33
MDPV	MDPV-d_8_	276.2	175.2	50	25
			205.2	50	25
Mephedrone	Mephedrone-d_3_	178.3	145.2	20	25
			160.3	20	16
Methadone	Methadone-d_3_	310.2	265.2	50	26
			105.1	50	37
Methamphetamine	Methamphetamine-d_5_	150.1	91.0	50	20
			65.0	20	54
Methcathinone	Methcathinone-d_3_	164.3	130.2	20	46
			146.2	20	16
Methylone	Methylone-d_3_	208.2	160.2	20	31
			132.3	20	34
Morphine	Morphine-d_3_	286.1	165.2	50	54
			153.2	50	50
Norbuprenorphine	Norbuprenorphine-d_3_	414.2	187.1	150	49
			211.2	150	49
Norfentanyl	Norfentanyl-d_5_	233.1	56.0	50	30
			84.1	50	23
Noroxycodone	Noroxycodone-d_3_	302.3	227.3	50	39
			284.2	50	23
Pholedrine	Pholedrine-d_3_	166.0	107.1	50	16
			135.0	50	18
PMA	MDMA-d_5_	166.3	121.2	20	31
			91.1	20	31
TFMPP	TFMPP-d_8_	231.3	77.2	100	46
			188.2	50	29
6-MAM-d_3_		331.2	165.2	110	35
Alpha PVP-d_8_		240.3	91.3	50	25
Amphetamine-d_5_		141.1	93.0	20	22
Benzoylecgonine-d_3_		293.1	171.2	50	27
Buprenorphine-d_4_		472.3	400.2	150	50
Cocaine-d_3_		307.3	185.2	50	25
Codeine-d_3_		303.1	165.2	80	54
Cotinine-d_3_		180.1	80.0	50	27
MDA-d_5_		185.1	168.1	20	14
MDMA-d_5_		199.1	165.1	20	15
MDPV-d_8_		284.2	134.3	50	31
Mephedrone-d_3_		181.3	148.2	20	20
Methadone-d_3_		313.2	268.2	50	26
Methamphetamine-d_5_		155.1	91.0	20	29
Methcathinone-d_3_		167.3	130.2	20	46
Methylone-d_3_		211.2	163.2	20	31
Morphine-d_3_		289.1	165.2	50	54
Norbuprenorphine-d_3_		417.2	187.1	150	49
Norfentanyl-d_5_		238.1	84.1	50	23
Noroxycodone-d_3_		305.2	287.2	50	23
Pholedrine-d_3_		169.0	107.0	50	16

a The first transition is the quantification transition, the second is the confirmation transition.

All data were acquired with Analyst 1.7 and processed using MultiQuant 3.0.2.

### LC–MS/MS optimisation

In this work, several chromatographic conditions were tested (i.e. flow rate, injection volume and mobile phase additives) to give the optimal conditions mentioned above.

All analysed compounds were tuned individually to give compound-specific declustering potential, collision energy and to find the most appropriate product ions for MRM analysis. For all of the compounds investigated, at least three product ions were monitored and the two most sensitive were selected to be the quantification (Q) and confirmation (q) transition (Table 1). The loss of water was avoided as the Q transition for any compound, based on its non-specificity, but in the case of methcathinone and mephedrone, it was chosen as the q transition due to the lack and/or insensitivity of other transitions. One transition was monitored for all of the internal standards. Each compound was quantified using its internal standard, with few exceptions (Table 1). For compounds without an analyte-specific ILIS, a surrogate ILIS was chosen based on its ability to correct for matrix effects and SPE losses.

In this work, both corrected (i.e. using ILIS) and uncorrected matrix effects and recovery were calculated as reported previously [7,13], utilising three sets of standards, spiked at 10 × LOQ: Set 1 (previously extracted SPE eluates spiked with the mixed standard and ILIS solutions), Set 2 (mixed standard solution in solvent, including ILIS) and Set 3 (SPE eluates spiked with the mixed standard and ILIS solutions prior to extraction):

$$\text{U}\text{n}\text{c}\text{o}\text{r}\text{r}\text{e}\text{c}\text{t}\text{e}\text{d}\;\text{m}\text{a}\text{t}\text{r}\text{i}\text{x}\;\text{e}\text{f}\text{f}\text{e}\text{c}\text{t}\text{s}:\frac{average\;peak\;area\;(Set\;1)}{average\;peak\;area\;(Set\;2)}\text{x}\;100$$

$$\text{C}\text{o}\text{r}\text{r}\text{e}\text{c}\text{t}\text{e}\text{d}\;\text{m}\text{a}\text{t}\text{r}\text{i}\text{x}\;\text{e}\text{f}\text{f}\text{e}\text{c}\text{t}\text{s}:\frac{\text{a}\text{v}\text{e}\text{r}\text{a}\text{g}\text{e}\;\text{p}\text{e}\text{a}\text{k}\;\text{a}\text{r}\text{e}\text{a}\;(\text{S}\text{e}\text{t}\;1,\;\text{I}\text{L}\text{I}\text{S})/\text{a}\text{v}\text{e}\text{r}\text{a}\text{g}\text{e}\;\text{p}\text{e}\text{a}\text{k}\;\text{a}\text{r}\text{e}\text{a}\;(\text{S}\text{e}\text{t}\;2;\;\text{I}\text{L}\text{I}\text{S})}{\text{a}\text{v}\text{e}\text{r}\text{a}\text{g}\text{e}\;\text{p}\text{e}\text{a}\text{k}\;\text{a}\text{r}\text{e}\text{a}\;(\text{S}\text{e}\text{t}\;1)/\text{a}\text{v}\text{e}\text{r}\text{a}\text{g}\text{e}\;\text{p}\text{e}\text{a}\text{k}\;\text{a}\text{r}\text{e}\text{a}\;(\text{S}\text{e}\text{t}\;2)}\text{x}\;100$$

Recovery and matrix effects were calucl Recovery was calculated using the following equations:

$$\text{U}\text{n}\text{c}\text{o}\text{r}\text{r}\text{e}\text{c}\text{t}\text{e}\text{d}\;\text{r}\text{e}\text{c}\text{o}\text{v}\text{e}\text{r}\text{y}:\frac{average\;peak\;area\;(Set\;1)}{average\;peak\;area\;(Set\;2)}\text{x}\;100$$

$$\text{C}\text{o}\text{r}\text{r}\text{e}\text{c}\text{t}\text{e}\text{d}\;\text{r}\text{e}\text{c}\text{o}\text{v}\text{e}\text{r}\text{y}:\frac{\text{a}\text{v}\text{e}\text{r}\text{a}\text{g}\text{e}\;\text{p}\text{e}\text{a}\text{k}\;\text{a}\text{r}\text{e}\text{a}\;(\text{S}\text{e}\text{t}\;1,\;\text{I}\text{L}\text{I}\text{S})/\text{a}\text{v}\text{e}\text{r}\text{a}\text{g}\text{e}\;\text{p}\text{e}\text{a}\text{k}\;\text{a}\text{r}\text{e}\text{a}\;(\text{S}\text{e}\text{t}\;2;\;\text{I}\text{L}\text{I}\text{S})}{\text{a}\text{v}\text{e}\text{r}\text{a}\text{g}\text{e}\;\text{p}\text{e}\text{a}\text{k}\;\text{a}\text{r}\text{e}\text{a}\;(\text{S}\text{e}\text{t}\;1)/\text{a}\text{v}\text{e}\text{r}\text{a}\text{g}\text{e}\;\text{p}\text{e}\text{a}\text{k}\;\text{a}\text{r}\text{e}\text{a}\;(\text{S}\text{e}\text{t}\;2)}\text{x}\;100$$

### Matrix effects

Matrix effects are recognised limitations of LC–MS/MS, which can lead to signal suppression or enhancement, and, in doing so, can adversely affect quantification. In complex matrices, such as influent wastewater, interferences can be particularly evident.

Uncorrected matrix effects show the true impact of the matrix on the analytes, with most showing signal suppression (Table 2). To correct for the suppression, ILIS were applied to give “corrected” matrix effects. The corrected matrix effects for all compounds except cotinine and morphine were good (100% ± 15%), which suggests that for these compounds some selective suppression was occurring.

**Table 2:** Method validation for all compounds in IWW including limits of detection and quantification, corrected and uncorrected matrix effects and absolute recovery and linearity. The values in brackets are the RSD (%).

Compound	LOD (ng/L)	LOQ (ng/L)	Uncorrected Matrix Effects (%)	Uncorrected Absolute Recovery(%)	Corrected Matrix Effects(%)	Corrected Absolute Recovery (%)	Linearity (range ng/L)
6-MAM	0.9	3	81 (4)	91 (4)	99 (12)	95 (12)	0.9968 (0.5–17.5)
Alpha PVP	0.2	0.6	62 (4)	101 (3)	118 (6)	110 (2)	0.9986 (0.5–21.5)
Amphetamine	3	10	35 (7)	111 (7)	114 (14)	122 (14)	0.9976 (2–425)
Benzoylecgonine	3	10	54 (3)	137 (4)	81 (4)	100 (4)	0.9984 (10–425)
Buprenorphine	0.3	2	48 (1)	99 (1)	83 (2)	107 (2)	0.9983 (2–425)
Cocaine	0.3	1	59 (3)	92 (4)	94 (2)	112 (2)	0.9999 (1–212.5)
Codeine	20	50	76 (15)	94 (15)	85 (20)	80 (20)	0.9997 (40–8500)
Cotinine	100	300	137 (17)	117 (16)	48 (8)	101 (8)	0.9996 (20–4250)
Ethylone	0.02	0.2	49 (4)	96 (4)	81 (9)	101 (9)	0.9969 (0.1–21.5)
MDA	0.7	2	64 (9)	134 (8)	82 (4)	83 (4)	0.9997 (2–275)
MDMA	3	10	54 (6)	96 (6)	92 (12)	94 (12)	0.9997 (10–275)
MDPV	0.3	0.8	57 (3)	98 (3)	86 (0)	110 (0)	0.9996 (0.5–17.5)
Mephedrone	1	5	66 (23)	85 (20)	83 (1)	133 (1)	0.9993 (0.2–35)
Methadone	0.3	1	42 (4)	102 (4)	82 (3)	108 (3)	0.9990 (1–212.5)
Methamphetamine	15	50	52 (3)	94 (3)	97 (11)	118 (11)	0.9995 (40–1700)
Methcathinone	0.8	2	44 (12)	121 (11)	95 (1)	118 (1)	0.9925 (1–35)
Methylone	0.3	1	44 (7)	90 (7)	90 (8)	109 (9)	0.9989 (0.1–21.25)
Morphine	30	100	94 (8)	104 (7)	55 (4)	101 (4)	0.9990 (20–4250)
Norbuprenorphine	5	10	47 (3)	101 (3)	86 (2)	107 (2)	0.9996 (2–350)
Norfentanyl	0.3	1	61 (8)	93 (7)	80 (6)	117 (6)	0.9995 (0.2–42.5)
Noroxycodone	0.4	1	64 (10)	102 (9)	82 (7)	103 (7)	0.9990 (1–212.5)
Pholedrine	3	10	47 (2)	111 (4)	106 (4)	91 (4)	0.9991 (8–1400)
PMA	0.7	1	46 (29)	90 (28)	110 (14)	119 (13)	0.9964 (1–21.25)
TFMPP	0.03	0.5	50 (4)	104 (4)	77 (15)	101 (14)	0.9993 (0.5–21.25)

LOD = limit of detection; LOQ = limit of quantification.

### Method validation

The method was validated in terms of linearity, limit of detection (LOD), limit of quantification (LOQ), accuracy and precision based on recovery experiments (Table 2). Linearity was examined with standard solutions in solvent at nine concentration levels, based on the projected LOQ of the analytes. Linearity was deemed satisfactory when the correlation coefficient was greater than 0.99 with all analytes passing.

LOD and LOQ were determined by spiking the standards into blank wastewater at concentration levels ranging from 0.01 to 300 ng/L to obtain a signal/noise ratio of >3 (LOD) and >10 (LOQ). Alternatively, for compounds which had endogenous levels, the LOQ was determined as being the lowest point on the calibration curve which gave a response within 20% of the spiked level [14]. The LOQs for the analytes tested ranged from 0.2 ng/L for ethylone to 300 ng/L for cotinine. The blank wastewater used for this experiment was collected on a Tuesday. This day of the week was chosen as stimulant and NPS consumption is typically lower mid-week [[15], [16], [17]], while the opioids analysed in this study are more habitually used and thus samples of all days of the week would have endogenous levels.

Precision and accuracy were evaluated using recovery experiments. All experiments were performed in triplicate. As seen in Table 2, all compounds had acceptable recoveries, proving the suitability of the applied SPE procedure for the analysis of these analytes. Overall, this SPE and LC–MS/MS method was validated for 24 analytes in wastewater. These included selected analytes of abuse potential which have wide-reaching interest in monitoring the health and illegal activity of communities. This method serves as the ideal starting point for future wastewater monitoring studies which aim to estimate critical substances of abuse potential.

## Additional information

Wastewater-based epidemiology (WBE) is rapidly becoming the technique of choice for monitoring the (illicit) drug consumption of a community, with reports for both the European Monitoring Centre for Drugs and Drug Addiction (EMCDDA; covering 56 cities and 19 countries in Europe) and the Australian Criminal Intelligence Committee (ACIC; covering up to 50 sites across Australia) routinely published [18,19]. Further monitoring programs have been implemented in New Zealand [20] and China [21]. Wastewater research has also been carried out in The Caribbean, Canada and the USA but to the best of the authors’ knowledge, no formal monitoring programs have yet been established.

The nation- or continent-wide monitoring programs traditionally focus solely on the consumption of illicit drugs, such as cocaine, ecstasy (MDMA and MDA), heroin, methamphetamine, amphetamine and cannabis. Several methods have been validated for these drugs [22,23]. However, our method included pharmaceutical opioids and substances with abuse potential to give a more comprehensive overview of drug consumption in a community in a more-timely manner.
